# Inflamed Eyelids

**Published:** 1891-05

**Authors:** 


### INFLAMED EYELIDS.

Eyes are too delicate and too valuable to be trifled with. One of their most frequent troubles is catarrhal ophthalmia, for which many simple alleviations and remedies are in use. In any affection of the eye one should not delay to seek medical advice. The front of the eyeball, and the surfaces of the eyelids which glide over the eye, are lined with a delicate membrane called the conjunctiva. Lying side by side in the substance of each eyelid, and connected with the conjunctiva, are the meibomian glands in parallel strings, about two dozen in each lid. The conjunctiva and meibomian glands are liable to the same congestive and inflammatory affection which in other mucous membranes is called a catarrh, or popularly a cold ; hence this affection when occurring in the eye is called catarrhal ophthalmia, or, popularly, a cold in the eye. For this the subjoined remedies may be safely tried ; but for inflammation of the subjacent sclero-cornea, or for anything else, a doctor should be consulted. These characteristics distinguish the two affections. First, the color of the inflamed conjunctiva ranges from red to bright scarlet, whereas the inflamed sclerotic varies from pink to almost purple. Next, the irregular network of vessels in the conjunctiva can be made to shift its place and slide about over the subjacent structures by merely dragging at the eyelids, whereas the more regular radiating vessels of the sclerotic are fixed, and keep their places under traction of the eyelids. And, lastly, the sensation of the conjunctiva is as of sand under the eyelids, or a fly in the eye, combined with dryness ; but in the sclerotic the pain is a dull aching and throbbing, combined with tightness. Now, if the sufferrer can satisfy herself that it is only her conjunctiva that is affected, with or without the meibomian glands, she may adopt these measures in the order in which they are given :—First, cold, strong tea used frequently as a lotion ; next, a lotion of dilute solution of acetate of lead, sometimes called Goulard water ; next, a solution of sulphate of zinc applied to the eye in such strength and frequency as the chemist who supplies it may direct. Nitrate of silver is not a thing to be lightly put in one's eye, nevertheless 4 grains of nitrate of silver in 1 oz. of distilled water, one drop of which is placed every few hours in the inner corner of the affected eye, is a favorite treatment of some physicians of high authority. In all cases apply glycerine or other fatty substance to the eyelids at night to prevent their being gummed together on waking. In cases of considerable pain use this prescription :—Hydrochlorate of cocaine, 8 grains; glycerine, 1 oz. ; boric acid, 3 drachms ; elder-water, 6 oz. ; water to 8 oz. ; a few drops in each eye two or three times daily. In a simple catarrh this treatment will be found effective.

